# Imagining the metaverse court: a conversation between science fiction and Shakespeare

**DOI:** 10.3389/fsoc.2025.1552706

**Published:** 2025-05-22

**Authors:** David Tait, Meredith Rossner

**Affiliations:** POLIS: The Centre for Social Policy Research, Australian National University, Canberra, ACT, Australia

**Keywords:** emotion, rituals, metaverse, courts, justice, virtual

## Abstract

This article explores the concept of a metaverse courtroom by engaging in an imaginative dialogue between Shakespeare's *Hamlet* and Neal Stephenson's *Snow Crash*. Using Connolly's method of juxtaposing distinct intellectual traditions, the analysis examines key aspects of justice processes—presence, facework, movement, adversarialism, and evidence presentation—in virtual spaces. Drawing on insights from dramaturgy, the sociology of emotions, and science fiction, the article considers how the performative and symbolic dimensions of physical courtrooms might translate to the metaverse. By imagining the metaverse courtroom as a space for innovation and interaction, this article seeks to illuminate how literature, sociology, and technology can collaboratively inspire the reimagining of justice in virtual environments.

## Introduction

This article introduces a thought experiment in which we bring together two quite different literary pieces and put them into an imaginary conversation about something new to both of them. It uses a method developed by political philosopher Connolly (2002), aimed at producing insights and innovations by drawing on literature to inspire scientific practice. The focus of this conversation how to design and run a courtroom in the metaverse. We organize the conversation around issues relevant to a justice process: managing spaces, organizing participants, producing respect, organizing an adversarial process and handling evidence.

The “metaverse” refers to an alternative world located online. It is an imaginary world: what participants see in front of them are simply pixels on a screen (or via a headset), arranged to mimic the continuous visual world. These configurations of pixels are converted into electrical signals by the retina of the eye and transmitted through the optic nerve to the brain, where they are interpreted as an image. The word “metaverse” was invented by Neal Stephenson in his 1992 science fiction novel, *Snow Crash*. The plot involves a virus (“snow crash”) that interrupts the process of transforming the pixels on the screen into an image—not only does the image on the screen turn into a field of snow, but the user's brain is also scrambled and the person starts talking nonsense. The story follows the hero (named Hiro Protagonist) as he seeks to rescue the world from a plague that is both physical and digital, with participants operating at the same time in two worlds—Reality and the Metaverse.

The other partner in the conversation, Hamlet, is a stage play in which the protagonist takes his time working out how to get justice for his father. There are many lessons it provides about staging, managing actors, and connecting with an audience. It also provides a creative approach to obtaining evidence and arranging equality of arms between combatants.

## Approaches to technological change

We have been interested in exploring the ritual, procedural, and symbolic implications of the use of virtual technology in justice spaces (authors' own). The shift to virtual technology in courts and tribunals is well documented, which presents a range of challenges and opportunities (Legg and Song, 2021; authors' own). While courts hearings that involve video technology are widely used, we anticipate the incorporation of more immersive virtual spaces. As such, we want to explore in this article what such a space could look and feel like. We call this space a metaverse courtroom. By metaverse we mean a collective, virtual shared space, created by the convergence of virtually enhanced physical reality and online environments, where users can interact, socialize, work, and play.

Developing a metaverse court could begin by modeling it after existing court hearings, whether traditional in-person settings or virtual courts like those using video conferencing software (e.g., “Zoom court”). Replicating essential features of physical courtrooms—such as lighting, acoustics, and participant spacing—can make the digital environment feel familiar to regular court users. Enhancements like elegant furnishings and wood paneling can add gravitas, while efficient processes for the movement of people and documents can improve functionality.

This digital reconstruction also offers an opportunity to address shortcomings of physical courtrooms. For instance, we can avoid confining defendants to docks or boxes that may imply guilt (see authors' own) and prevent witnesses from feeling isolated (Rowden et al., 2013). Adjustments like modifying the judge's bench height, improving sightlines between participants, and incorporating symbolism that reflects contemporary values can enhance the courtroom experience.

While video conferencing technology might increase access to justice by reducing travel costs and limiting unwanted encounters between parties, it has limitations. In a “zoom court” where participants appear on a screen “gallery style,” they often cannot make eye contact, appear in separate boxes with varied backgrounds, and experience sound from a single source, making it hard to identify speakers. The absence of a shared environment can hinder the development of empathy and the exercise of authority, enhance vulnerabilities and impact the assessment of witness credibility (Bandes and Feigenson, 2021; McKay and Macintosh, 2024).

A metaverse platform can address these issues by placing participants in a shared virtual space where they can make apparent eye contact and receive directional sound cues. In our initial development of an immersive virtual court—which achieved eye contact through multiple cameras—we found that the realism of the hearing matched that of face-to-face settings. However, the authority of the judge and the credibility of witnesses were rated significantly lower (authors' own).

An incremental approach to metaverse courtrooms—making gradual technical adjustments—aligns with how innovation often develops. For example, it took nearly a thousand years to advance from block printing in ninth-century China to Stanhope's cast-iron printing press in 1800. However, another strategy takes inspiration from literary sources, particularly science fiction, which imagines the future before it happens (Michaud, 2017; Michaud and Appio, 2022; Bucher, 2019). When law engages with technology, Tranter (2011, p. 817) argues “science fiction is its speculative jurisdiction.” Extending this principle to literature or the arts more generally, several literary forms can be brought into conversation with one other.^1^ This approach recognizes that innovation may result from networks of information rather than just a single source (Hargadon, 2003).

This synergistic approach resonates with Connolly's (2002) idea of bringing together distinct “tributaries” of knowledge—different fields, thinkers, or practices—into conversation. Connolly, as a political philosopher, juxtaposes perspectives to explore questions like the “ethics of cultivation.” For example, he combined Foucault's analysis of authority and surveillance, Nietzsche's focus on performing ideas, and Derrida's emphasis on media, showing how each thinker corrects and complements the others.

Connolly extends this idea further by connecting philosophy, neuroscience (examining brain and body processes behind intellectual ideas), and film (expressing ideas through performance and emotion). His aim is not to predict the future but to make its possibilities “shine more brightly” (Connolly, 2002, p. 22). By engaging the imagination, he creates new ways to explore and understand potential futures, even if they don't materialize.

Inspired by this approach, we return to the original conceptualization of the metaverse, originating in science fiction. We also engage with a more ancient literary approach to creating justice, *Hamlet*. By bringing together these two unlikely sources, we hope to draw out the key concepts and elements of a metaverse court.

## Science fiction as inspiration for technological change

The first “tributary” to be considered, *Snow Crash*, operates within a genre that has long been associated with scientific discovery. Science fiction has long inspired technological innovation and anticipated future developments (Jordan et al., 2018). While writers like H.G. Wells, Jules Verne, and Isaac Asimov are often credited with influencing inventions such as liquefied rocket fuel, submarines, and driverless cars (Winter, 2008; Poluhowich, 1999; Höltgen, 2025; Asimov, 1968),^2^ it is Mary Shelley who stands as one of the earliest and most influential figures in the genre.

Shelley, immersed in both science and literature, drew from the works of leading scientists like Erasmus Darwin, Benjamin Franklin, and Humphrey Davy (Freedman, 2002), as well as her exposure to Shakespeare, particularly *Hamlet* (Shelley, 1992). Part of a circle that included Percy Shelley and Lord Byron, she famously wrote *Frankenstein, or The Modern Prometheus* (1818), a story born from a shared challenge to write ghost stories. Her tale explored the dangers and possibilities of pushing scientific boundaries by creating intelligent life, foreshadowing ideas of artificial intelligence and the concept of computers (Patowary, 2023). In this, Shelley exemplifies how science fiction can mobilize networks of knowledge—science, literature, and philosophy—to generate new ways of thinking about technology and its consequences. This is a vivid example of the synergistic approach later outlined by Connolly.

## The two sources

We propose to use an approach similar to both Shelley and Connolly, bringing together art and social science to imagine justice in the metaverse. We start with science fiction—the genre that Shelley contributed to developing—providing a close reading of *Snow Crash* by Neal Stephenson, the science fiction story that, as noted above, introduced the concept of the metaverse, as well as bringing to life the concept of the avatar.

We put this into conversation with Shakespeare's *Hamlet: Prince of Denmark* (Jenkins, 1982). On the surface these have little to say to each other. One is a stage play, a revenge tragedy, about a prince seeking to avenge his father's death by killing the uncle who poisoned his father and then married his mother. Meanwhile *Snow Crash* is a novel, a satirical form of cyberpunk, a warning about how the US may collapse into anarchy. One is a finely polished work of art that is widely thought to have perfected an age-old story by giving all the characters depth and complexity. The other is a speculative venture into a technology that was about to emerge in which the characters are lightly sketched in. However, we are not comparing their genre, character development, major themes or contribution to world literature. We are putting them into a conversation about issues that are specifically relevant to justice processes: how presence and participation is established, the role of face and demeanor, how the setting and participants interact with each other and are managed, and how evidence is produced and presented.

Stephenson's (1992) *Snow Crash* is a satirical vision of a dystopian future in which the US is fragmented into a range of warring fiefdoms controlled by greedy tycoons, while the environment teeters on the brink of collapse. To escape this bleak reality, people turn to the metaverse, an immersive virtual world Stephenson invents and names. Borrowing the term “avatar” from the Vedic scriptures, he populates the metaverse with digital identities who inhabit their own plots of virtual land connected by “The Street,” a boulevard that extends around the virtual world.

The story follows Hiro Protagonist, a freelance hacker living on society's margins, and YT, his 15-year-old courier friend who often bails him out of trouble. The plot centers on a mysterious virus, *Snow Crash*, which uniquely bridges the digital and physical worlds. The virus damages the brains of individuals who interact deeply with computers—effectively synchronizing their minds with machine operating systems. Others are infected by the virus through an addictive drug or contaminated blood provided by a radical Pentecostal church.

As the digital and physical realms become increasingly intertwined, Hiro uncovers the truth about the virus and its origins. In the climactic finale, one of the main oligarchs—who controls the fiber-optic infrastructure of the Metaverse—is killed, two others are gravely injured, and those infected by *Snow Crash* are freed from its grip.

Hamlet is one of Shakespeare's most celebrated tragedies. Like the dystopian world of *Snow Crash*, the world is “out of joint.” Here, the disruption stems from the untimely death of Hamlet's father, the rightful king, whose brother Claudius murders him by pouring poison into his ear. Claudius seizes the throne and marries Hamlet's mother, Gertrude, compounding the disorder.

Haunted by his father's ghost, who urges him to avenge the murder, Hamlet grapples with doubt, moral uncertainty, and the burden of duty. After a period of hesitation and confirming Claudius's guilt, Hamlet finally kills the usurper in a final scene of chaos and violence, leaving nearly all the central characters dead.

This analysis brings together these two “tributaries”—Hamlet and Snow Crash, to reimagine justice in the metaverse. Both sources, though separated by centuries and genres, offer powerful insights into the dynamics of presence, authority, and interaction, making them compelling foundations for conceptualizing virtual courtrooms. Additionally, we incorporate relevant computer games and films that address related issues or offer further perspectives or practical illustrations of how the metaverse might function. We also draw on scholarship from the sociology of emotions and performance, literatures that we have argued are particularly well-suited to provide insights into designing and researching virtual courts (authors' own). Concepts such as facework, dramaturgy, and framing are particularly relevant to understanding how participants interact, manage impressions, and negotiate authority in both physical and virtual settings. By applying these concepts to the metaverse, we aim to highlight the social and emotional dimensions of virtual justice, which are often overlooked in technologically focused discussions. This includes an analysis of the management of presence and visibility, the staging and choreography of movement and interaction, and the role of performative elements in establishing authority and legitimacy in court settings.

## Being there: double presence

A metaverse courtroom fundamentally reshapes our understanding of presence in legal proceedings. Unlike traditional courtrooms where participants occupy a single, shared physical space, metaverse environments enable what we call “double presence”—the ability to simultaneously exist and operate in multiple spaces. This dual existence creates new possibilities for how participants engage with the court, access information, and interact with others. Both *Snow Crash* and *Hamlet* offer unique perspectives on this concept, demonstrating how individuals can navigate between public and private domains, physical and virtual realms, while maintaining meaningful engagement in both.

Virtual reality games aim to give players the experience of “being there,” an illusion of presence enhanced when the environment and other players provide confirmatory cues (Heater, 1992; Lombard and Ditton, 1997). The goal of the games designer, as with a filmmaker, is to get the participants or audience to suspend disbelief, to become fully immersed in the fiction created for their entertainment. While metaverse courts similarly aim to create a convincing experience of “being there” they also need to ensure that participants are able to operate fully in their local environment. This double presence (authors' own) allows judges and lawyers, to engage fully with participants or documents both in local and remote locations.

In *Snow Crash* participants live in two realities. There is Reality, as it is called, which is chaotic and violent. Some people live in this world in comfortable surroundings, such as gated communities, protected by bodyguards; Hiro lives in a storage shed. The second reality is the metaverse, a virtual realm accessed through computers, goggles, and a shared fiber-optic network. Hiro's interactions often occur in both spaces at once, blending Reality and the Metaverse:

“*Where are you?” Hiro says. “In Reality or the Metaverse?” “Both. In the Metaverse, I'm on a plusbound monorail train ....” “Where are you in Reality?” “Public terminal across the street from a Reverend Wayne's,” she says*. (p. 233)

In *Snow Crash*, people move fluidly between virtual and physical spaces, though nobody entirely forgets their place in Reality. While *Snow Crash* predates immersive platforms like Second *Life* or *World of Warcraft*, its depiction of double presence resonates with later explorations of digital interaction. Notably, the Metaverse in *Snow Crash* remains imperfect—freezing and re-rendering under strain—reminding us of the technological limitations that must be considered.

Both worlds are deeply unequal, reflecting social divides. Reality is dominated by warlords and oligarchs, while ordinary people are marginalized and need to align themselves with a powerful patron to survive, for instance Hiro works in a mafia-run pizza franchise. Similarly, in the metaverse technological disparities determine access. Hiro enjoys well-developed avatars in full color with advanced tools. Others have to use a public access sites, appearing as low-resolution black-and-white renderings.

In *Hamlet*, Shakespeare similarly presents two worlds. There is the visible, physical world experienced by the play's characters—Elsinore Castle and its surroundings—and a hidden world, accessible only to Hamlet and his confidants, where the ghost of his father reveals unsettling truths. Hamlet operates across seven primary physical settings, including public and private spaces: the battlements, royal chambers, private chapels and closets, and a graveyard. A pirate ship—not shown on the stage—rescues Hamlet. Additionally, Shakespeare introduces a “stage within a stage” through the *play within a play*, expanding the layers of presence and performance.

Hamlet and select friends have access to a spirit world, where the ghost of Hamlet's father embodies this dual presence, appearing in both visible and hidden spaces. It gestures for Hamlet to follow it to secure privacy, recalls an orchard where Claudius poisoned him, and later speaks from *beneath* the stage, urging secrecy. The ghost disrupts the boundaries of space and presence, much like digital interventions in the Metaverse.

A metaverse court allows for similar movement. Judges and lawyers could shift between physical sidebars and virtual meeting rooms, or hearings could occur in dynamic environments, such as virtual pirate ships or open orchards inspired by *Snow Crash* and *Hamlet*.^3^

Participants might operate simultaneously in public and private spaces, much like Hiro and YT's layered conversations. Public hearings could occur in physical or virtual courtrooms, while private deliberations take place backstage or in parallel virtual spaces. Drawing on Hamlet's ghost—appearing from unexpected locations—participants could “disrupt” proceedings to emphasize accountability or moral obligation, adding performative layers to court processes.

The 2024 film *Grand Theft Hamlet* demonstrates the potential—and challenges—of staging performances in a metaverse environment. Attempting to perform *Hamlet* within the open-access game *Grand Theft Auto*, two actors face an unanticipated challenge: random audience members enter the virtual space, disrupting the play by killing the actors' avatars. This “play within a play” parallels Shakespeare while highlighting the unpredictability of virtual settings.

This experiment reveals the emerging maturity of metaverse platforms for hosting dramatic, emotional interactions—key components of justice processes—while warning that unplanned disruptions remain a risk. A metaverse courtroom, like *Hamlet*'s stage, would require careful design to balance formal procedures with flexibility, ensuring justice remains at the center of the performance.

## Facework: self-presentation, authenticity, authority

The effectiveness of justice processes depends heavily on the management of identity, authority, and respect among participants. Drawing on Goffman's concept of facework (Goffman, 1967)—the strategies individuals employ to present themselves and maintain dignity in social interactions—we can better understand how participants in a metaverse court might navigate their self-presentation through avatars while preserving the authority and legitimacy essential to judicial proceedings.

In a metaverse court, the face can take on a new meaning with the introduction of avatars. Avatars can serve as digital extensions of a participant's identity, but they also require careful design to convey authority, respectability, and authenticity. With this comes a new “frame”—new sets of cues guiding participants in how to act, how to feel, and how to interpret the behavior of others (Goffman, 1974). In both *Hamlet* and *Snow Crash*, characters engage in negotiations of identity and authenticity, offering insights into how faces and facades operate in contexts where traditional social cues are disrupted or transformed.

In *Hamlet*, the struggle to maintain face in a fractured environment contributes to much of the play's tension. The political and moral order of Denmark is “out of joint,” and Hamlet repeatedly challenges the self-presentation of others, exposing their hypocrisies and hidden truths. His interactions are often laced with mockery, undermining the public personas others strive to maintain.

For instance, Hamlet humiliates Polonius by pretending to mistake him for a “fishmonger” and accusing him of dishonesty, mockery that escalates when Hamlet casts aspersions on Polonius's daughter's chastity and age (Act 2, Scene 2). The ultimate affront comes when Hamlet kills Polonius, saying, “I took thee for thy better,” a cold comfort for the man mistaken for the king.

Hamlet's attack on face—literal and metaphorical—extends to his love interest Ophelia in his comment:

“*God hath given you one face, and you make yourself another.”*

On the surface, this line critiques women's use of cosmetics, referring to the lead-based makeup popular in Elizabethan England. Scholars often interpret it as a misogynistic jibe (Mullaney, 1994), but within the broader context of the play, Hamlet's statement connects to themes of artifice and deception. Claudius, for instance, earlier delivers a striking self-condemnation, likening his “painted words” to a prostitute's painted face:

“*The harlot's cheek, beautied with plastering art, Is not more ugly to the thing that helps it Than is my deed to my most painted word.”* (Act 3, Scene 1)

Claudius's metaphor acknowledges his guilt, hidden beneath a veneer of polite words, while Hamlet's critique of Ophelia's “two faces” reveals a similar frustration—men, seduced by superficial appearances, are complicit in their own self-deception. Hamlet's subsequent reflection on his own offenses (“more offenses at my beck than I have thoughts…”) suggests that his critique of Ophelia is as much self-directed as it is accusatory. This duality—holding others accountable while revealing his own failings—demonstrates Hamlet's awareness of the performance inherent in self-presentation.

Hamlet operates within layers of performance: public and private, visible and hidden. He exposes the painted faces of others while struggling with his own dual identity—one for the court, one for his inner self. This conflict culminates in soliloquies and asides, spaces where Hamlet can temporarily drop the mask and express his true thoughts to the audience. Lawyers in contemporary court cases can adopt the opposite strategy, putting on a “stoneface” to hide their true feelings, distancing themselves from their clients (Flower, 2018, p. 124). Such a practice is also common amongst judges (Bergman Blix and Wettergren, 2018).

In *Snow Crash*, facework takes on a new dimension in the Metaverse, where avatars replace physical selves. Unlike Hamlet's world—where face reflects dignity and authenticity—avatars are human creations that can be endlessly modified and recreated. This fluidity both empowers and destabilizes identity.

Hiro Protagonist reflects on the significance of face when he meets a receptionist daemon who matches his ethnicity:

“*If a white man had stepped off the elevator, she probably would have been a blonde.”* (p. 449)

Here, face is not authentic but adaptive, designed to match the expectations of the viewer. Hiro himself admits to underestimating the importance of facial expressions when coding avatars. While he focused on body movements, his colleague Juanita developed the crucial facial coding that enables avatars to convey emotion and nuance:

“*Nobody thought that faces were all that important… She was just in the process of proving them all desperately wrong.”* (pp. 65–66)

Hiro later critiques the male-dominated coding culture for failing to recognize the significance of face, paralleling Hamlet's self-reflection on men's complicity in superficial judgments.

In the Metaverse, avatars also challenge conventional ideas of dignity and permanence. Sword fights, for example, may leave avatars humiliated—“red-faced and sweating”—but such failures are fleeting. Dead avatars are quickly cleared, and participants can log back in with renewed faces. As Hiro observes, avatars can appear differently to different viewers, adapting their appearance as needed.

The tension between authentic and constructed faces in *Hamlet* and *Snow Crash* has clear implications for a metaverse courtroom. In virtual justice settings, participants might manage their avatars to present different faces depending on the audience or context. For example:

- A defendant might appear as a neutral, featureless avatar or might present a sympathetic face.
- Avatars could be programmed with gestures and movements that simulate respectful interactions—such as bowing or nodding when addressing the judge—to reduce the risk of disinhibition often associated with virtual spaces.
- Judges might shift between stern avatars for formal hearings and approachable visages for mediation.
- Audience members could opt for ghost-like anonymity or avatars that signal group affiliations or messages.

This adaptability mirrors the layered performances in *Hamlet*, where public and private faces coexist, and participants manage their self-presentation for different audiences. It also reflects the ephemeral nature of facework in *Snow Crash*, where avatars can be reset or customized as needed. The question, then, is whether a metaverse court should encourage participants to reveal their “true” faces or allow them to construct strategic avatars.

Ultimately, a metaverse courtroom could embrace the dynamic potential of avatars, allowing participants to switch between public and private “faces,” much like Hamlet navigating Elsinore or Hiro shifting identities in the Metaverse. By rethinking facework, virtual courts could balance the performance of authority with spaces for authentic dialogue and emotional expression.

Goffman reminds us that maintaining face involves strategic and collaborative acts, tied to social cues and demands of a particular encounter. In *Hamlet's* court and the metaverse of *Snow Crash*, faces are not fixed; they adapt to shifting frames and contexts, requiring effort and intentionality to uphold. A metaverse courtroom, drawing from these insights, would need to account for the strategic, relational, and performative nature of face. Avatars and their mutable appearances could enrich interactions by providing opportunities to express authority and authenticity, acknowledge the emotional complexity of justice, and foster respectful engagement. Face in a metaverse court takes work to achieve but opens up new possibilities for rethinking identity, interaction, and authority in justice spaces.

## Movement, choreography, and staging

Movement and spatial organization are integral to the choreography of justice in physical courtrooms. From the formal entrance of the judge to the positioning of witnesses and defendants, these movements and arrangements communicate power, authority, and procedural structure. A metaverse courtroom presents both challenges and opportunities for reimagining this choreography, allowing for new forms of movement, interaction, and spatial design that may enhance or transform traditional court dynamics. *Hamlet* and *Snow Crash* both feature distinctive approaches to movement and staging that can inform how we conceptualize navigation and interaction in virtual justice spaces.

### Journeying to court

In physical courtrooms, movement is an integral part of the proceedings, shaping the flow of interactions, reinforcing authority, and maintaining the formality of the space. Judges enter the courtroom to the call of “All rise,” lawyers stand and sit as needed, witnesses take their turn in the stand, and defendants often remain seated, observing the process. Lawyers might walk over to confer with clients, place a reassuring hand on a shoulder, or pause to sip water. Members of the public shuffle in and out, monitored by attendants. These physical cues and movements choreograph the courtroom's rhythm, creating a sense of order and structure.

In a metaverse courtroom, replicating these physical interactions raises challenges. For instance, in *Snow Crash*, avatars avoid physical contact, as the absence of tactile feedback “reminds you that you're not even really there” (p. 253). However, some actions, such as serving virtual drinks or receiving a shoulder rub, enhance the immersive illusion. Carefully designed protocols—like ensuring avatars cannot pass through walls or materialize arbitrarily—reinforce the metaphor of presence:

“*You can't just materialize anywhere in the Metaverse, like Captain Kirk beaming down from on high. This would be confusing and irritating to the people around you. It would break the metaphor.”* (p. 43)

Currently, virtual justice platforms like Zoom rely on participants “beaming in” and “out,” a convenience that sacrifices realism. Introducing more deliberate movements—such as walking or teleporting into the courtroom—could elevate the experience, grounding participants in a shared virtual space. Considering the experience of appearing at court consisting of a “journey” to the courtroom can enhance the solemnity and legitimacy of proceedings (authors' own).

### Managing people: movement and ghosts

In *Hamlet*, choreography reflects the interplay between public and private worlds. Characters frequently move across different spaces—public chambers, private closets, and the outdoor graveyard—mirroring their shifting roles and relationships. Movement often emphasizes power dynamics: Claudius delivers commands from the audience chamber, Hamlet soliloquizes in isolation, and confrontations unfold in intimate settings like Gertrude's closet.

The characters in *Snow Crash* are constantly on the move, often using vehicles like scooters, motorcycles, helicopters, and trucks. Even in the Metaverse, Hiro's hypersonic motorbike highlights how movement retains symbolic and practical importance, even without physical constraints. Navigation reinforces presence and interaction, making the virtual world feel immersive and dynamic rather than static or disconnected. Movement, real or virtual, is central to engaging with the world and asserting one's role within it.

Coordinating group movement within a metaverse is particularly challenging. In *Snow Crash*, avatars in public spaces like The Street often walk through each other, a pragmatic solution to the technical demands of rendering multiple avatars simultaneously. However, this disrupts the illusion of physical presence:

*On the Street, avatars just walk right through each other… When things get this jammed together, the computer simplifies things by drawing all of the avatars ghostly and translucent so you can see where you're going*. (p. 47)

Private spaces like the Black Sun club offer a more realistic experience. Here, avatars are rendered solid, collisions are prevented, and access is limited to ensure smooth interaction:

“*In The Black Sun, avatars are not allowed to collide. Only so many people can be here at once, and they can't walk through each other.”* (p. 63)

In *Hamlet*, the ghost of Hamlet's father further complicates staging, appearing in both visible and invisible forms. Sometimes the ghost is seen only by Hamlet, while at other times its presence is indicated through sound effects, such as knocking from beneath the stage. These techniques blur the boundaries between physical and metaphysical spaces, offering inspiration for managing presence and interaction in a metaverse courtroom.

A metaverse courtroom could adopt similar strategies, designating areas with varying levels of immersion or realism. For instance, the well of the court could operate like the Black Sun—solid and orderly—while public gallery spaces could adopt the translucency of The Street to accommodate higher numbers of avatars.

### Staging soliloquies and asides

*Hamlet* employs soliloquies and asides, creating layers of engagement. Soliloquies allow characters to speak directly to the audience, revealing inner thoughts and motivations, while asides provide confidential commentary amid public scenes. These devices enhance the audience's understanding while maintaining the illusion that other characters remain unaware.

### Application to a metaverse courtroom

Drawing inspiration from *Snow Crash* and *Hamlet*, a metaverse courtroom could innovate movement and staging in ways that enhance both functionality and symbolism. Avatars could be rendered invisible or translucent when not actively participating, much like ghostly figures on The Street in *Snow Crash* or hidden characters in *Hamlet*. Protected witnesses could also be made visible only to judges and lawyers, maintaining anonymity while enabling selective interaction. Audio could be layered to allow private sidebars between lawyers or confidential exchanges with clients, echoing whispered conversations in physical courts or asides in *Hamlet*.

Participants might move “onstage” or “offstage” depending on their role, with soliloquy-like “backstage” opportunities for defendants or witnesses to share reflections privately with the judge or jury (Goffman, 1959). Judges could summon participants into the well of the court for specific interactions, recreating the formal entrances and exits typical of physical courtrooms. Virtual spaces could also be adapted to suit different proceedings, ranging from realistic courtroom settings to more symbolic environments, such as an amphitheater for public deliberations or a private chamber for mediation.

Incorporating these elements into a metaverse courtroom requires balancing technical feasibility with the symbolic and performative aspects of justice. *Snow Crash* highlights the importance of managing group dynamics and maintaining the illusion of presence, while *Hamlet* demonstrates the power of staging and movement to convey authority, conflict, and reflection. Together, these sources suggest that a metaverse court need not simply replicate physical courtrooms but can reimagine justice as a dynamic, layered performance, blending visibility, movement, and interaction to create a series of spaces where participants are both present and empowered.

## Adversary and action

A defining feature of many courtroom processes is the adversarial structure, where two sides confront each other to present their case and challenge the evidence of their opponents. In English common law, this tradition originates in the accused's right to physically “confront” their accuser, a principle that evolved into a contestation of words. Modern trials are typically managed by professional lawyers, who engage in verbal duels through oral arguments, written submissions, and the examination and cross-examination of witnesses.

This adversarial tradition is deeply rooted in the historical practice of trial by combat, where disputes were settled, and “truth” determined through physical confrontation. Although trial by combat largely disappeared after the Fourth Lateran Council of 1215 prohibited clergy from participating in such practices, its language and metaphors endure in contemporary courts. Lawyers are said to “attack” or “ambush” witnesses, “confront” discrepancies in testimony, or engage in “onslaughts” during cross-examination. Even the concept of giving both sides “equality of arms” harks back to the days when physical weapons decided justice.

A metaverse court offers new possibilities for reimagining how adversarial processes might function when freed from physical constraints while still maintaining procedural fairness. Both *Hamlet* and *Snow Crash* feature confrontations and conflicts that illuminate different dimensions of adversarial interaction, suggesting innovative approaches to balancing opposition with fairness in virtual environments.

In *Hamlet*, adversaries clash with both words and swords. Hamlet himself acknowledges the potency of words as weapons: “*I will speak daggers to her, but use none.”* Yet he often resorts to literal violence, as when he impulsively stabs Polonius through a curtain or leaps onto Ophelia's grave to fight her brother, Laertes. Ultimately, the play culminates in a duel between Hamlet and Laertes, orchestrated by the manipulative Claudius, which ends in the deaths of nearly all the main characters.

In *Snow Crash*, Hiro engages in combat both physically and virtually. As the programmer who wrote the Metaverse's sword-fighting code, he is undefeated in his virtual battles, culminating in a final confrontation with Raven, the novel's antagonist, in which Hiro triumphs by decapitating him. Unlike Hamlet, Hiro survives his adversarial encounters, but both characters navigate worlds where conflict is inevitable, and the stakes are high.

In a courtroom context, physical combat is replaced by verbal argumentation, but the adversarial structure persists. A metaverse courtroom might extend this tradition by offering new ways to embody or visualize these confrontations. For instance, the principle of “equality of arms” could be reinforced by placing opposing parties at the same level, facing each other, as seen in Scandinavian and German courtrooms. By contrast, the common law practice of seating both parties at the same table might undermine the visual metaphor of an adversarial contest.

The metaverse also allows for innovative ways to immerse participants and audiences in the dynamics of a trial. For example, as in *Hamlet*, soliloquies and asides provide the audience with privileged access to characters' thoughts and motivations. A metaverse court could replicate this through immersive first-person perspectives, allowing jurors or judges to momentarily “step into the shoes” of different participants. This could provide a deeper understanding of conflicting viewpoints by letting the audience experience the trial from multiple perspectives. For the disputants, new rituals and a “just distance” between the parties could encourage them to recognize the humanity of the other (Ricoeur, 1995; Garapon, 1997).

While traditional courtroom proceedings rely on verbal exchanges, a metaverse court could incorporate visual and interactive elements to make these interactions more engaging. Lawyers and judges already use visual aids in trials; expanding this to include three-dimensional reconstructions or immersive experiences would be a natural progression. For example, participants might use avatars to visually demonstrate arguments or evidence, creating a more dynamic and memorable experience for jurors.

Despite these possibilities, any metaverse courtroom would need clear rules to ensure fairness and preserve the adversarial nature of the process. As seen in *Hamlet* and *Snow Crash*, conflicts are most compelling when the playing field is level. In the metaverse, this could mean controlling how participants present themselves and interact, ensuring that all parties have equal access to tools and representation. Allowing one side to manipulate the rules or gain an unfair advantage, as Laertes does with his poisoned sword or as Hiro avoids through his coding expertise, would undermine the legitimacy of the process.

The comparison between *Hamlet, Snow Crash*, and modern courts highlights both the drama and limitations of adversarial proceedings. While courtroom disputes are rarely as action-packed as sword fights or virtual battles, the metaverse offers an opportunity to bridge this gap by combining the narrative power of storytelling with immersive technologies. By integrating dynamic visuals, first-person perspectives, and equitable staging, a metaverse courtroom could transform the adversarial contest into a more engaging and accessible experience while staying true to its foundational principles.

## Gathering and presenting evidence

A key feature of contemporary trials is the identification, presentation, and assessment of evidence. Evidence can take the form of written documents, oral testimony, or increasingly, video and audio recordings. Regardless of its format, evidence must be rigorously tested to confirm its relevance and reliability. This fundamental process is mirrored in both *Hamlet* and *Snow Crash*, where the protagonists adopt creative strategies to uncover and verify truth.

In *Hamlet*, the titular character initially receives evidence about his father's murder through oral testimony, albeit from a ghost. Recognizing the need to test the ghost's account, Hamlet devises an innovative approach: he commissions actors to perform a play mirroring the alleged murder of his father, complete with the distinctive detail of poison poured into the king's ear.

…*I'll have these players* *Play something like the murder of my fatherBefore mine uncle: I'll observe his looks;I'll tent him to the quick: if he but blench*,*I know my course…*

Hamlet's goal is to provoke a reaction from Claudius, whose guilt would confirm the ghost's testimony. To maximize the play's impact, Hamlet instructs the actors to “hold, as 'twere, the mirror up to nature,” creating an authentic performance designed to elicit an emotional response. The strategy works: Claudius storms out during the poison scene, betraying his guilt and validating Hamlet's suspicions. This moment demonstrates how an interactive and realistic simulation can bring hidden truths to light.

However, not all evidence-gathering techniques in *Hamlet* are equally effective. The final duel between Hamlet and Laertes, sanctioned as a formal trial by combat, fails to yield a satisfactory resolution. Both combatants die, as does the king, leaving the dispute unresolved. This outcome reflects historical critiques of trial by battle as an unreliable means of discovering truth.

Meanwhile, Hamlet himself becomes the subject of evidence-gathering. Claudius deploys a network of spies to determine whether Hamlet knows of his father's murder and is plotting revenge. Hamlet's feigned madness serves as a strategy to obscure his true intentions, buying him time to develop his plans. This interplay of surveillance and deception highlights the complexity of evidence collection, where motives and interpretations can be clouded by misdirection.

In *Snow Crash*, Hiro's evidence-gathering mission parallels Hamlet's, though it is driven by technology rather than theater. Tasked with uncovering the origins of a deadly virus, Hiro relies on an AI assistant called the Librarian to access and compile vast amounts of information. The Librarian traces the virus's origins to ancient Sumer, its spread through neurolinguistics and Pentecostal Christianity, and its suppression by linguistic diversity following the Tower of Babel. Despite its speed and breadth of knowledge, the Librarian admits its limitations:

*The connections are elaborate. Summarizing them would require both creativity and discretion. As a mechanical entity, I have neither*. (p. 245)

Hiro must interpret and synthesize this information himself, demonstrating that even advanced AI cannot fully replicate human judgment or creativity. Unlike Shelley's Frankenstein's monster, the Librarian does not evolve into a self-aware entity but remains a tool dependent on human agency.

Both *Hamlet* and *Snow Crash* show that evidence-gathering requires not only tools and techniques but also human insight to interpret and act on the information. These lessons have direct implications for the design of a metaverse courtroom. Advanced AI systems, akin to Hiro's Librarian, could assist in processing and organizing evidence, while immersive simulations could mirror Hamlet's play within a play, allowing participants to test and visualize allegations. In addition, AI could facilitate language interpretation, enabling seamless communication between participants speaking different languages, or help construct dynamic re-enactments to examine evidence from multiple perspectives.

In a metaverse court, these tools could enhance the accessibility and reliability of evidence, but their use must be carefully balanced with human oversight. Just as Hamlet's performance relied on Claudius's reaction to confirm its validity, and Hiro's investigation depended on his ability to connect disparate threads, evidence in a metaverse court must ultimately be scrutinized and evaluated by human participants. The integration of AI and simulations offers immense potential, but it also demonstrates the enduring necessity of creativity, discretion, and critical thinking in the pursuit of justice.

## How issues facing video courts might apply to metaverse courts

The purpose of this Article is not to provide a blueprint for improving current ways of operationalizing virtual courts but, following Connolly, to unsettle established knowledge and challenge assumptions about what (in this example) justice processes might look like. This could be to take advantage of the opportunities offered by metaverse technologies, or more broadly re-think justice rituals and spaces. Nevertheless, to make some of the ideas more concrete it is useful to sketch out briefly how some of the issues associated with virtual courts using video conferencing technology might apply to metaverse courts.^4^

### Accessibility

Accessing a video hearing typically requires a basic computer with an adequate broadband link. Taking part in a metaverse court hearing requires more expensive gear: a gaming computer with a fast CPU and a high-quality graphics card. This could be a barrier for potential users. Such technology might be made available in communication pods within public facilities like town halls, public libraries or lawyers' offices (as well as facilities within courthouses). Some of Hiro's colleagues had access to such a facility even if their avatars appeared in a simpler form. Providing such infrastructure requires more planning and investment than allowing people to take part in a video court hearing from a smartphone in a car or from a tablet in their bedroom. But insisting on minimum standards for the remote environment is likely to provide greater privacy for the user, fewer distractions for others in the hearing, more dignity and less chance of network dropouts. Such communication pods could of course be also used for video hearings. For people living in regional and remote areas either technology would provide enhanced accessibility compared to driving long distances to physical courtrooms.

### Procedural fairness

Fairness is a central concern in any justice hearing. Equality of arms was the fairness principle violated in Hamlet's final sword fight with Laertes. In both the video court and the metaverse court ensuring the defense has equally good access to suitable technology will be the challenge. The presumption of innocence meanwhile is routinely violated for defendants in physical courts in the UK, Canada, France, and most Australian states by placing them in docks, even sometimes in glass cages (author's own). Showing the accused on a screen alongside their lawyer can avoid this problem (author's own). Effective access to counsel can be an issue in any form of distributed hearing, but if client and lawyer are co-located (whether in a courtroom or elsewhere) this can enable effective communication between them, even if it makes it more difficult for the two parties to communicate. Metaverse courts would be no different in this respect. Vulnerable witnesses meanwhile can be intimidated or even placed in danger if they are visible during a hearing. This is solved in international tribunals by pixelating the vulnerable witness being video streamed; a metaverse court addresses the problem differently by having everyone appear as avatars, drawing less attention to the form of the witness. If a virtual jury trial is held (as some were during the pandemic), it is hard for jurors to get spatial and audio cues if the faces of the active participants are framed in boxes in a gallery looking forward. A metaverse court would provide a more comprehensive approach by showing all the (other) participants embedded in a shared environment.

### Security

Occasionally video hearings have been disrupted by outsiders engaging in what has been dubbed “Zoom bombing,” not unlike what happened in *Grand Theft Hamlet*. Most video conferencing systems now have multiple layers of security so even if random visitors do manage to break into a virtual hearing (which is usually public anyway), they can usually enter only the video streaming room (without participant privileges) or if they venture further, they can be quickly excluded by the presiding officer. Metaverse courts pose more of a security threat—gaming platforms can be used as a back end for malicious users to gain access to a computer network. For this reason, metaverse applications (whether for courts or anything else) tend to require the very high security environments of cloud servers.

### Empathy

Reduced possibilities for empathy is one of the limitations identified for video hearings (Bandes and Feigenson, 2021). It is possible that seeing the avatar of a person is even less likely to evoke empathy than seeing the person's video image in a video hearing. On the other hand it is expected that seeing fuller bodily gestures from participants framed within a courtroom setting rather than a Zoom gallery, observing interactions including how others respond to a statement and feeling part of a shared environment—these together could counter any disadvantage associated with seeing a person in the form of an avatar. Even if a metaverse setting or the avatar appearance has little observable impact on empathy, it might improve the ability of decision makers to listen carefully to the evidence without distraction, something that is arguably key to producing just outcomes from a judicial process. These are empirical claims that could be tested by experimental studies.

### Inclusiveness and reduction in intimidation

Garfinkel's critique of some (in-person) courtroom interactions as degradation rituals does suggest that the bar for inclusiveness in justice processes is fairly low (Garfinkel, 1956). Courts are not places where people generally feel comfortable. The bar is potentially even lower for remote participants who at present rarely get a comprehensive view of the court. They are in effect visually excluded, seeing only the judge and lawyers, so are limited in their ability to assess the impact of their presence or their evidence. This is not a flaw inherent in the technology, indeed it is entirely possible for the witness or defendant to see the audience, jury (if there is one) and both parties, which in experimental studies has been shown to have a positive impact on the satisfaction of the remote participant (Rowden et al., 2013). In a metaverse court on the other hand active participants would all see and be seen throughout the hearing as if they were physically co-present (albeit in avatar form). While the number of audience members who can be shown is restricted by bandwidth, live streaming of hearings for both video courts and metaverse courts can allow for larger audiences than traditional face-to-face hearings. For people who come from a stigmatized minority or have body image issues being able to choose an avatar that they feel comfortable with could make a court appearance feel less threatening and more welcoming.

### Efficiency and cost

Metaverse platforms would be more expensive to run than virtual courts using video conferencing. Apart from the higher cost associated with fast graphics cards within the computers used, higher quality cameras and monitors would be required to take advantage of the technology, and a secure server would be essential. Any efficiencies in cost of running courts would likely be derived from transfer of hearings from physical hearings to either form of online hearing. Where a metaverse platform has already begun to lead to real savings is in the design of physical court spaces—architects can simulate alternative configurations and allow their clients to identify optimal sightlines, distances, and relative heights.

### Privacy

Virtual courts during the pandemic faced several privacy challenges. Live streaming of cases could potentially allow viewers to secretly record and distribute selected extracts from open hearings, regardless of any judicial warnings. Meanwhile vulnerable participants (such as in mental health hearings) might have personal information about them disclosed to random strangers (Scarlett, 2020). Adding password entry procedures, court participants retiring to breakout rooms for confidential discussions, and using initials rather than names were introduced as a way of reducing the risk of breach of privacy. In relation to privacy from inappropriate audience activity a metaverse court could face similar risks and could adopt similar safeguards. However, any form of open court could allow disclosure of embarrassing information, such as unseemly conflict over a family trust or uninhibited statements made during a night of revelry. Streaming a hearing, whether of a video or a metaverse court, could increase the risk of mass dissemination of what court users might consider private information. A metaverse court could shield participants from visual exposure, while pseudonyms could reduce the chances of identification.

### Adaptability to contemporary legal needs

Whether metaverse justice hearings could replace other hearing modes for some types of case on some occasions is an empirical question. Complex commercial cases where parties and witnesses are dispersed seem particularly suitable. Evidentiary hearings before criminal trials might be another possible application, including when proposed evidence is in a three-dimensional form (such as a virtual walk-through of a crime scene). Most companies offering video conferencing systems are already developing more immersive environments with the goal of supporting various forms of virtual co-presence, so the difference between a video image and a high quality avatar's appearance is likely to become increasingly narrow.

## Conclusions

In exploring how a metaverse court might function, we have drawn from the seemingly disparate worlds of *Hamlet* and *Snow Crash*. This dialogue of perspectives, inspired by Connolly's method of juxtaposing intellectual tributaries, has revealed new ways to conceptualize justice as both a practical and performative process. Each text contributes unique insights into key courtroom dynamics—presence, face, movement, adversarialism, and evidence—while offering complementary critiques of their limitations.

At its core, the metaverse court challenges us to move beyond static, traditional notions of justice spaces. The concept of “double presence,” exemplified by both Hamlet's ghost and Hiro's dual realities, suggests that participants can operate in layered spaces—public and private, real and virtual—offering a richer, more dynamic understanding of engagement. This layering reflects not only the spatial hybridity of the metaverse but also the emotional and social complexities of a justice encounter. Managing one's role, dignity, and interactions in relation to others—concepts central to facework and framing—becomes essential to maintaining both authority and authenticity in virtual justice environments.

The performative insights of *Hamlet* resonate in this context. Hamlet's soliloquies and asides offer a model for creating private, reflective spaces in a public courtroom setting, while the “play within a play” demonstrates how re-enactments can reveal hidden truths. Similarly, *Snow Crash* shows the importance of movement and presence in virtual settings, as seen in Hiro's hypersonic motorbike journeys. The embodied actions of avatars—whether navigating between spaces, adopting different forms, or engaging with evidence—become a vital part of asserting agency and fostering interaction. Indeed the forms of interaction that may develop in the new environment may go beyond simply replicating traditional practices, they are likely to create new practices (Dumoulin and Licoppe, 2009).

This analysis reminds us that justice spaces are deeply emotional and interactive spaces (Bergman Blix and Wettergren, 2018). Shelley's *Frankenstein* exemplifies the blending of science and art to explore the ethical and emotional dimensions of creation. Her work provides a reminder that technological spaces, including the metaverse, must grapple with the social and emotional consequences of the worlds they generate.

The documentary *Grand Theft Hamlet* serves as both an inspiration and a cautionary tale. It demonstrates the potential for metaverse spaces to host emotionally charged, dramatic interactions that resonate deeply with participants and audiences. At the same time, it highlights the risks of unplanned disruptions and the challenge of maintaining order in open, interactive environments.

By bringing these sources into conversation, we are enabled to see the metaverse court as more than a replication of physical proceedings let alone an enhanced version of the “Zoom court.” It emerges as potentially a layered, flexible, and performative space. Or rather “spaces”—participants can move between a range of customized locations, with adjustable visibility settings. Faces can be “painted” according to either court conventions or individual choice. Following Connolly's invitation, designing a metaverse court is not about predicting the future but making its possibilities vivid, tangible, and meaningful. It is possible that the conversation would have taken a different turn if we had included a different set of sources. The risks of technological innovation might have assumed greater prominence if we had followed Mary Shelley's creation further down its path of self-destruction. Or we might have been less accommodating of judicial authority if we had used Spartacus as our model rather than Hamlet. However, the frame that Connolly provides of juxtaposing two apparently unrelated literary pieces acts to unsettle “normal science” and linear assumptions about progress. Just as Hamlet's ghost disrupts both the emotional security of the play's protagonist and the assumption of a two-dimensional stage, so the device of bringing diverse sources together allows us to disrupt the way we think about metaverse courts.

## Footnotes

^1^Science fiction is not of course the only form of knowledge that can be argued to have inspired innovation. A similar argument has been made for mathematics, for example in the way perspective in Renaissance art directly drew on Indian mathematics, transmitted through the Arabic world. See Dalrymple (2024).

^2^Not all technological advances foreshadowed in science fiction are cast as beneficial. Ray Bradbury's *Fahrenheit 451* in 1953 foresaw a future in which the population spent much of their time mindlessly watching television. Add a camera to a TV set and in George Orwell's *1984*, the devices could be used to watch the citizenry. Smartphones, a prototype of which appeared in Star Trek, can be used for not only to monitor people and track their movements. As well as raising questions about the dangers posed by new technologies, science fiction has provided a platform for examining the social order of the present, by transferring the issues to a distant planet or unfamiliar environment. This might involve the “colonial gaze” in which the imperial adventures of European powers are made more palatable by being transferred into the colonization of distant planets inhabited by savages, or the transformation of Captain James Cook, the British explorer, into Captain James Kirk of the *Starship Enterprise*. It could be more overtly political as in HG Wells' parable about the genocide of Australian Aboriginal peoples in *War of the Worlds*, or indeed the critique of anarcho-capitalism—a world torn apart by oligarchs and faced with climatic disaster–in *Snow Crash*.

^3^The idea of designing a building to reference a pirate ship comes from another visual source, a factory in Berlin in the Steiner system that supports people with disabilities. See https://www.feddersen-architekten.de/portfolio/werkstatt-fuer-behinderte-in-berlin-zehlendorf/.

^4^We are grateful to an anonymous reviewer for suggesting this section, and identifying the issues that should be examined.
